# Optimizing the Use of Phase Change Material Vests Worn During Explosives Ordnance Disposal Operations in Hot Conditions

**DOI:** 10.3389/fphys.2020.573521

**Published:** 2020-10-29

**Authors:** Sarah Lee Davey, Ben James Lee, Mark Smith, Mark Oldroyd, Charles Doug Thake

**Affiliations:** ^1^Occupational and Environmental Physiology Group, Centre for Sport, Exercise and Life Sciences (CSELS), Faculty of Health and Life Sciences, Coventry University, Coventry, United Kingdom; ^2^United Shield International, Andover, United Kingdom

**Keywords:** extreme environments, heat stress, uncompensable heat stress, explosive ordnance disposal, phase change cooling

## Abstract

Phase change material (PCM) cooling garments’ efficacy is limited by the duration of cooling provided. The purpose of this study was to evaluate the effect of replacing a PCM vest during a rest period on physiological and perceptual responses during explosive ordnance disposal (EOD) related activity. Six non-heat acclimated males undertook three trials (consisting of 2 × 3 × 16.5 min activity cycles interspersed with one 10 min rest period) in 40°C, 12% relative humidity whilst wearing a ≈38 kg EOD suit. Participants did not wear a PCM cooling vest (NoPCM); wore one PCM vest throughout (PCM1) or changed the PCM vest in the 10 min rest period (PCM2). Rectal temperature (T_*re*_), mean skin temperature (T_*skin*_), heart rate (HR), Physiological Strain Index (PSI), ratings of perceived exertion, temperature sensation and thermal comfort were compared at the end of each activity cycle and at the end of the trial. Data displayed as mean [95% CI]. After the rest period, a rise in T_*re*_ was attenuated in PCM2 compared to NoPCM and PCM1 (−0.57 [−0.95, −0.20]°C and −0.46 [−0.81, −0.11]°C, respectively). A rise in HR and T_*skin*_ was also attenuated in PCM2 compared to NoPCM and PCM1 (−23 [−29, −16] beats⋅min^–1^ and −17 [−28, −6.0] beats⋅min^–1^; −0.61 [−1.21, −0.10]°C and −0.89 [−1.37, −0.42]°C, respectively). Resulting in PSI being lower in PCM2 compared to NoPCM and PCM1 (−2.2 [−3.1, −1.4] and –0.8 [−1.3,−0.4], respectively). More favorable perceptions were also observed in PCM2 vs. both NoPCM and PCM1 (*p* < 0.01). Thermal perceptual measures were similar between NoPCM and PCM1 and the rise in T_*re*_ after the rest period tended to be greater in PCM1 than NoPCM. These findings suggest that replacing a PCM vest better attenuates rises in both physiological and perceptual strain compared to when a PCM vest is not replaced. Furthermore, not replacing a PCM vest that has exhausted its cooling capacity, can increase the level of heat strain experienced by the wearer.

## Introduction

Wearing personal protective equipment (PPE) in hot environments and/or during high intensity activity can result in the body being unable to maintain thermal balance, which is termed as uncompensable heat strain (UHS; see Cheung et al., 2000 for a review). Due to the nature of the role, explosive ordnance disposal (EOD) operators wear fully encapsulating armor suits that provide blast and fragmentation protection for the whole body with the most vulnerable body parts (i.e., head, torso, face) being prioritized for protection. The mass (>35 kg) and impermeable nature of these suits make operators susceptible to significant metabolic strain and UHS (Stewart et al., 2011, 2013) which can lead to reduced work tolerance (Stewart et al., 2014), symptoms of heat related illnesses (Stewart et al., 2013) and the possibility of reducing cognition, particularly on complex tasks that involve executive functioning and memory (Racinais et al., 2008; Gaoua et al., 2011); all of which can negatively affect the health and safety of the operator.

When UHS occurs during operation, it is not always feasible to loosen or remove clothing or to reduce the work rate. Therefore, several cooling strategies have been developed and demonstrated to reduce or attenuate the level of heat strain experienced whilst wearing encapsulating protective clothing resulting in extended tolerance times and/or favorable thermal perceptions (McLellan et al., 1999; Cadarette et al., 2003; Kenny et al., 2011; Watkins et al., 2018; Bach et al., 2019; Maley et al., 2020). One such cooling strategy is personal microclimate cooling garments. Several types of cooling garments exist, i.e., air-cooled garments, liquid-cooled garments, and phase change material (PCM) garments. When body surface area (BSA) coverage is similar, liquid and air-cooled garments generally provide superior cooling capacity and more favorable physiological responses to that of PCM garments when wearing PPE in hot environments or during high metabolic activities (Chan et al., 2015). However, due to their complexity, heavy mass, restriction in mobility, and the cost to procure and maintain liquid and air-cooled garments, PCM garments are generally utilized in the field (Chan et al., 2015).

Phase change material absorbs the body heat carried to the surface of the skin with additional heat energy taken up, with no change in temperature, by the phase transition from a solid to a liquid. Phase change materials come in several forms, such as ice-based and substances that melt at a greater temperature, i.e., long-chain alkanes, such as hexa- and tetra-decane. The latter substances have the advantage of being able to be recharged (solidified) without a freezer (e.g., a PCM that melts at 24°C can be recharged in water or air below 24°C). The main drawback of PCM garments, and hence its inferior cooling capacity in comparison to liquid and air cooled garments, is that PCM is no longer effective as a method of cooling when the PCM’s temperature has passed the phase change range especially when used during operation as a method of per cooling (Ying et al., 2004). In addition, if the cooling capacity has become negligible, the additional weight, insulation and/or impermeable layer and possible movement restriction could increase the level of heat strain due to an increased metabolic rate or a reduction in heat dissipation. Therefore, the time period of a PCM’s effectiveness can be limited (McLellan and Frim, 1998; Reinertsen et al., 2008; Maley et al., 2020), especially in higher ambient temperatures or at higher metabolic rates where a curvilinear relationship exists between tolerance time and metabolic rate during uncompensable heat stress (McLellan et al., 1993). As a consequence, currently PCM cooling garments are recommended for use in operations that last 1–2 h depending upon the body surface area covered, PCM melting temperature and the mass of PCM garment (McLellan and Frim, 1998; Gao et al., 2010; Chan et al., 2015).

One solution to this problem is to replace or recharge the PCM in the garment once the PCM’s temperature has passed the phase change range. In the field EOD personnel often have a rest period after ≈45 min. This rest “window” potentially provides an opportunity for the PCM to be replaced. Previous studies have assessed the effectiveness of certain personalized cooling garments where the cooling method (i.e., PCM or ice-based) is recharged during use (i.e., per cooling), however, they do not compare the effectiveness of this recharge against a control condition (Muir et al., 1999; Cadarette et al., 2003; Butts et al., 2017), therefore, the efficacy of this approach is unknown. To the authors’ knowledge the effect of replacing or recharging a PCM garment during operation on both thermal physiological and perceptual responses has not been investigated during uncompensable thermal stress. Therefore, the aim of this exploratory study was to determine the physiological and perceptual responses to replacing PCMs garments during a simulated EOD work scenario whilst wearing an EOD suit in a hot environment.

## Materials and Methods

### Participants

Six males participated in the study (age: 22 ± 3 years; body mass: 78.1 ± 9.3 kg; height 176.3 ± 6.5 cm). All participants were verbally briefed, issued with a participant information sheet, and gave written informed consent. All were non-heat acclimated, non-smokers and as indicated by the completion of a PAR-Q Health Screen Questionnaire had no history of illness (cardio-respiratory or metabolic disease). Ethical approval for the procedures was obtained from the Coventry University ethics committee and designed in accordance with the Declaration of Helsinki (2013) regarding human experimentation.

### Experimental Procedures

**FIGURE 1:**
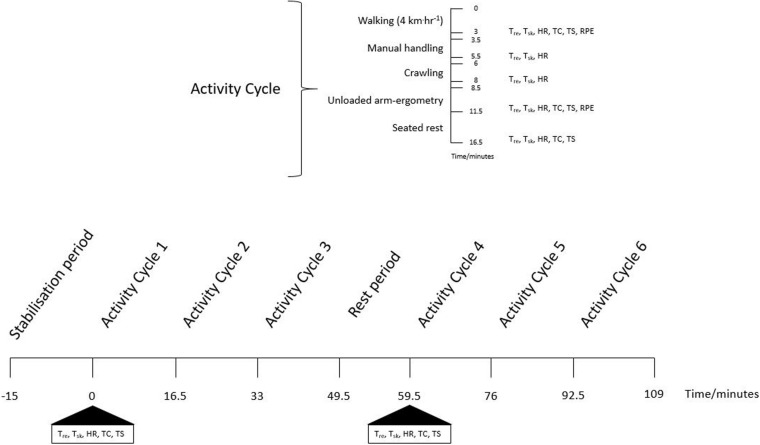
A schematic diagram of the experimental procedures outlining the activities that occurred in each activity cycle and timings of key measurements. T_*re*_, rectal temperature; T_*sk*_, skin temperature; HR, heart rate; TC, thermal comfort; TS, temperature sensation; RPE, rate of perceived exertion. Measurements were taken within each activity cycle and before and after the stabilization and rest periods. Measurements from the end of unloaded-arm ergometry were compared between cycles. In the measurements of HR and Physiological Strain Index, the average from all data points within an activity cycle were compared between cycles. End of trial measurements were those collected at the of the rest period in activity cycle 6.

After completing a familiarization session, all participants undertook three trials (each separated by at least 7 days) that involved completing a work/rest regime whilst wearing a ≈38 kg EOD suit (Mk6, NP Aerospace, United Kingdom) in 40.3 ± 1.4°C, 12 ± 1.7% relative humidity (rh) and in two of the trials a phase change material (PCM) cooling vest (Jackson Technical Solutions Ltd., Norfolk, United Kingdom). Each trial consisted of an initial 15 min stabilization period at 20°C where participants rested in a seated position wearing a cotton t-shirt, combat trousers and combat boots. After which participants donned the EOD ensemble, over the undergarments, and if applicable the PCM cooling vest, prior to entering the climate chamber. The cooling vest was worn over the cotton t-shirt. The time period between the end of 20°C stabilization period and entering the environmental chamber, including donning the EOD suit, was kept constant between trials within participants. In the climatic chamber, participants completed up to 2 × 3 (16.5 min EOD specific activity cycles designed to replicate the physical tasks that operatives encounter in the field interspersed with one 10 min rest period, taken after the first 3 activity cycles; 109 min in total) (Thake et al., 2009). Each activity cycle consisted of 3 min of walking (4 km⋅h^–1^); 2 min of manual handling consisting of moving four 1.25 kg weights between two shelves, one 64 cm the other 27 cm from the floor to the beat of a metronome (30 beats⋅min^–1^, Seiko DM 20, Hattori Seiko Co., Ltd.; Japan) whilst kneeling; 2 min of crawling and searching activity comprising of a pre-defined pattern of moving forward and back along a 2.25 m “ladder,” marked on the environmental chamber floor and right and left “searching” head turns with movements entrained to a metronome (30 beats⋅min^–1^); 3 min period of seated unloaded arm-ergometry at 60 rev⋅min^–1^; and 5 min of seated rest. A standardized 30 s time transfer time was included between the walking, manual handling, and crawling and searching activities. The 10 min rest period took place after the completion of 3 activity cycles where the helmet, shield, jacket and PCM cooling vest, if worn, were removed and donned in first and last 90 s, respectively (Figure 1). In a randomized cross over design the three trials differed by participants not wearing the PCM (NoPCM); wearing one PCM cooling vest throughout the trial (PCM1) or the PCM cooling vest being replaced by a unused cooled PCM cooling vest (PCM2) during the 10 min rest period. Trials were completed at the same time of day for each participant (either 10 am or 2 pm) to account for circadian rhythm changes in core temperature and cardiovascular responses.

The familiarization session was completed by participants 1 week prior to commencing their first experimental trial. The familiarization session involved participants completing four of the EOD activity cycles used in the experimental trials whilst wearing the Mk6 EOD suit in 30°C, 30% rh. No PCM cooling vest was worn during the familiarization trial.

### Clothing System

In each trial participants wore 100% cotton standard combat trousers, T-shirt and leather steel toed boots under the EOD suit. All participants wore the Mk6 EOD suit (NP Aerospace, United Kingdom), which included a helmet, jacket, groin shield, trousers, and internal dual fan cooling system. The dual fan cooling system delivered ∼200 L⋅min^–1^ of ambient air to the wearers back and ∼100 L⋅min^–1^ to the head area throughout. In trials PCM1 and PCM2 participants wore a PCM cooling vest that covered the participants’ torso (back and chest) and had a mass of 1.12 kg. The PCM had a melting point of 25°C, and was thus solid below 25°C. A melting point of 25°C was chosen in consideration of the likelihood of being able to cool the vest to below 25°C in a hot environment akin to environmental conditions experienced in the Middle East.

The PCM cooling vest was cooled in a commercial fridge set to 5°C for in excess of 3 h prior to usage. The recommend commercial guidelines (as instructed by NP Aerospace Ltd., United Kingdom) stated the time required to “charge” the PCM material was no less than 30 min.

### Measurements

The following measurements were recorded in all trials: (1) rectal temperature (T_*re*_: self-inserted 10 cm past the anal sphincter; Grant Instruments; Cambridgeshire, United Kingdom); (2) local skin temperature (lateral calf, medial thigh, upper arm and chest) using steel mounted skin thermistors (Grant Instruments; Cambridgeshire, United Kingdom); these local skin temperatures were used to calculate mean skin temperature (T_*skin*_; Ramanathan, 1964); and (3) heart rate (Polar Vantage, Finland). At baseline, during the rest periods and in the final 30 s of treadmill walking and arm ergometry participants were asked for their subjective ratings of thermal sensation (TS) and thermal comfort (TC). Only in the final 30 s of treadmill walking and arm ergometry ratings of perceived exertion (RPE) were recorded. A 15 point scale (6–20; Borg, 1970) was used to monitor the participants overall RPE. Overall thermal sensation (Young et al., 1987) and thermal comfort (modified from Epstein and Moran, 2006) were rated by participants using a 9 point scale (0–8).

### Calculations

Notwithstanding the limitations of the traditional two-compartment thermometry model to estimate change in mean body temperature during exercise (Jay et al., 2007), heat storage (HS) was calculated using the equation by Havenith et al. (1995):

$$\begin{array}{c} \mathrm{HS}=\left(0.8\times \left[\text{Tre}\left(\text{t}\right)-\mathrm{Tre}\left(0\right)\right]\right)+\left(0.2\times \left[T_{skin}\left(\text{t}\right)-T_{\mathrm{skin}}\left(0\right)\right]\right)\times 3.49 \end{array}$$

where T_*re*_(t) = current T_*re*_, T_*re*_(0) = initial T_*re*_, T_*skin*_(t) = current T_*skin*_ and T_*skin*_(0) = initial T_*skin*_, 3.49 = the specific heat of body tissue (J⋅g^–1^⋅°C^–1^)

The Physiological Strain Index (PSI) was calculated using the equation by Moran et al. (1998a):

$$\mathrm{PSI}=5\times \frac{\text{Tre}\left(\text{t}\right)-\mathrm{Tre}\left(0\right)}{39.5-\mathrm{Tre}\left(0\right)}+5\times \frac{\mathrm{HR}\left(t\right)-\mathrm{HR0})}{180-\mathrm{HR}\left(0\right)}$$

where T_*re*_(t) = current T_*re*_, T_*re*_(0) = initial T_*re*_, HR(t) = current HR and HR(0) = initial HR.

Overall perception based strain index (PeSI; 0–10) was calculated using the equation by Tikuisis et al. (2002) the RPE and TS scores were altered from 15 (6 to 20) and 9 (0 to 8) point scales to 11 (0 to 10) and 7 (7 to 13) point scales, respectively.

$$\mathrm{PeSI}=5\times \frac{\text{TS}\left(\text{t}\right)-7}{6}+5\times \frac{\mathrm{RPE}\left(t\right)}{10}$$

where TS(t) = current TS and RPE(t) = current RPE.

Sweat rate (SR) was calculated using the following calculation:

$$\mathrm{SR}=\frac{\begin{array}{c} {}[\mathrm{Nude}\mathrm{body}\mathrm{mass}\left(\mathrm{pre}-\mathrm{trial}\right)\\ -\mathrm{Nude}\mathrm{body}\mathrm{mass}\left(\mathrm{post}\mathrm{trial}\right)]+\\ {}[\mathrm{Volume}\mathrm{of}\mathrm{fluid}\mathrm{consumed}\text{-}\mathrm{Volume}\\ \mathrm{of}\mathrm{water}\mathrm{excreted}] \end{array}}{\mathrm{Time}\left(h\right)}$$

### Statistical Analysis

All continuous data are presented as means and 95% confidence intervals (CI), and ordinal data are presented as median and interquartile range. Data were tested for normality using the Shapiro-Wilk test. A general linear model two-way repeated measures analysis of variance (ANOVA: condition: 3, time: 6–8) was used to compare T_*re*_, T_*skin*_, HS, HR, and PSI recorded at baseline, 30 s before the end of the arm ergometry exercise during each activity cycle and at the end of a trial for the majority of measures. To truly represent HR and PSI during an activity cycle mean HR and PSI across an activity cycle were compared between conditions across the six activity cycles. Ordinal data (RPE, TC, TS, and PeSI) were assessed for skewness and kurtosis, and were each found to display a normal distribution, so were analyzed via parametric general linear model two-way repeated measures ANOVA as described above. TC and TS were compared between conditions at baseline and 30 s before the end of the arm ergometry exercise during each activity cycle. RPE and PeSI were compared between conditions during arm ergometry across the six activity cycles. Significant (*p* < 0.05) main effects for condition, time and condition × time interaction were investigated using Tukey *post hoc* tests and corrected for multiple comparisons. Where statistically significant interactions were identified, simple main effects comparing the three experimental conditions across a given time point were performed, and Tukey corrected *p*-values reported. Effect sizes for main effects and interactions are reported as partial eta squared, whereas effect sizes for Tukey *post hoc* tests were calculated using an unbiased Cohen’s d (*d*_*unb*_) (Cumming, 2012).

$$\mathrm{Unbiased}{\mathrm{Cohen}}^{\prime }sd=\left(1-\frac{3}{4\left(\mathrm{df}-1\right)}\right)\times \left(\frac{\text{Mdiff}}{\text{SDav}}\right)$$

$$\mathrm{SDav}=\sqrt{\frac{{\mathrm{SD1}}^{2}+{\mathrm{SD2}}^{2}}{2}}$$

Where df is the degrees of freedom, Mdiff is the difference in means between two trials, and *SD1* and *SD2* are the *SD* of the two trials. All statistical procedures were performed using the Statistical Package for the Social Sciences 25.0 for Windows (SPSS, Inc., Chicago, IL, United States).

## Results

One of the participants was unable to complete the PCM1 trial and stopped prematurely after the treadmill exercise in cycle five due to fatigue. Therefore, statistical analysis to assess the interaction between condition and time were completed on the physiological and subjective responses of the five participants who completed all trials. Due to technical error and excessive measurement artifacts, one of the subjects HR recordings has been removed from the analysis, reducing the HR and PSI sample size to four. In addition, it was observed that the PCM cooling vest had changed from solid to liquid by the start of the 10 min rest period (i.e., after cycle three, ∼50 min of activity) in both PCM1 and PCM2.

### Physiological Measurements

**FIGURE 2:**
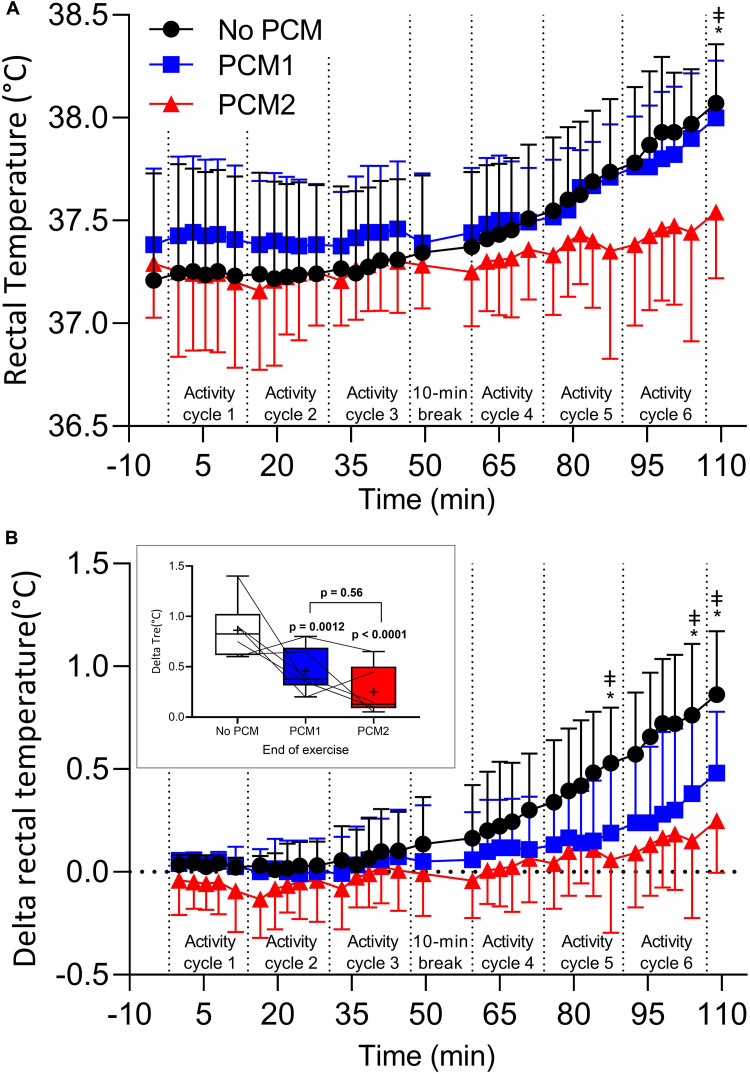
Rectal temperature **(A)**, delta rectal temperature **(B)**, recorded at baseline and 30 s before the end of the each exercise during an activity cycle, 10 min cooling period and 5 min rest periods. Data are the mean values ± 1 *SD*. Unidirectional error bars have been used to maintain clarity. To further illustrate differences between the different PCMs, the inset shows delta changes in rectal temperature at the end of the final activity cycle. Box plots display individual data points (lines), the 25 and 75th interquartile ranges (boxes), the median (mid-line), and mean group response (cross). Whiskers illustrate the highest and lowest value. Measures recorded at baseline, during arm ergometry at the end at the end of each 16:30 min:sec EOD activity cycle and at the end of a trial were compared between conditions (*N* = 5). *, difference between NoPCM and PCM2 (*p* < 0.05); ‡ = difference between NoPCM and PCM1 (*p* < 0.05).

There was a main effect for time in T_*re*_ [*F*(7, 28) = 34.77, *p* < 0.001, η*_*p*_*^2^ = 0.897] and for an interaction between condition and time in T_*re*_ [*F*(14, 56) = 2.91, *p* = 0.020, η*_*p*_*^2^ = 0.421], however, no main effect for condition [*F*(2, 8) = 2.16, *p* = 0.178, η*_*p*_*^2^ = 0.351]. *Post hoc* pairwise comparisons identified that T_*re*_ in PCM2 was lower than NoPCM (by −0.57 [95% CI: −0.95, −0.20]°C, *p* < 0.001, *d*_*unb*_ = 1.37) and PCM1 (by −0.46 [95% CI: −0.81, −0.11]°C, *p* = 0.0002, *d*_*unb*_ = 1.20) at the end of the trial. No other differences were established between conditions across the time points (Figure 2A). When T_*re*_ was expressed as delta change from rest, there was a main for time [*F*(6, 24) = 39.02, *p* < 0.0001, η*_*p*_*^2^ = 0.907] and for an interaction between condition and time [*F*(12, 48) = 3.23, *p* = 0.0019, η*_*p*_*^2^ = 0.447], however no main effect for condition was found [*F*(2, 8) = 1.72, *p* = 0.238, η*_*p*_*^2^ = 0.301]. *Post hoc* pairwise comparisons identified that ΔT_*re*_ in PCM1 was lower than NoPCM at the end of cycle 5 (by –0.35 [95% CI: −0.61,−0.09]°C, *p* = 0.005, *d_*unb*_* = 1.05), cycle 6 (by –0.40 [95% CI: −0.66, −0.14]°C, *p* = 0.0012, *d_*unb*_* = 0.93), and at the end of exercise (by –0.40 [95% CI: −0.66,−0.14]°C, *p* = 0.0012, *d_*unb*_* = 1.13). The ΔT_*re*_ was also lower in PCM2 compared to NoPCM at the end of cycle 5 (by –0.39 [95% CI: −0.65,−0.13]°C, *p* = 0.0017, *d_*unb*_* = 1.19), cycle 6 (by –0.54 [95% CI: −0.80, −0.28]°C, p < 0.0001, *d_*unb*_* = 1.35) and the end of exercise (by –0.51 [95% CI: −0.77, −0.35]°C, *p* < 0.0001, *d_*unb*_* = *1.46*). No differences were identified between PCM1 and PCM2 for ΔT_*re*_ (Figure 2B).

**FIGURE 3:**
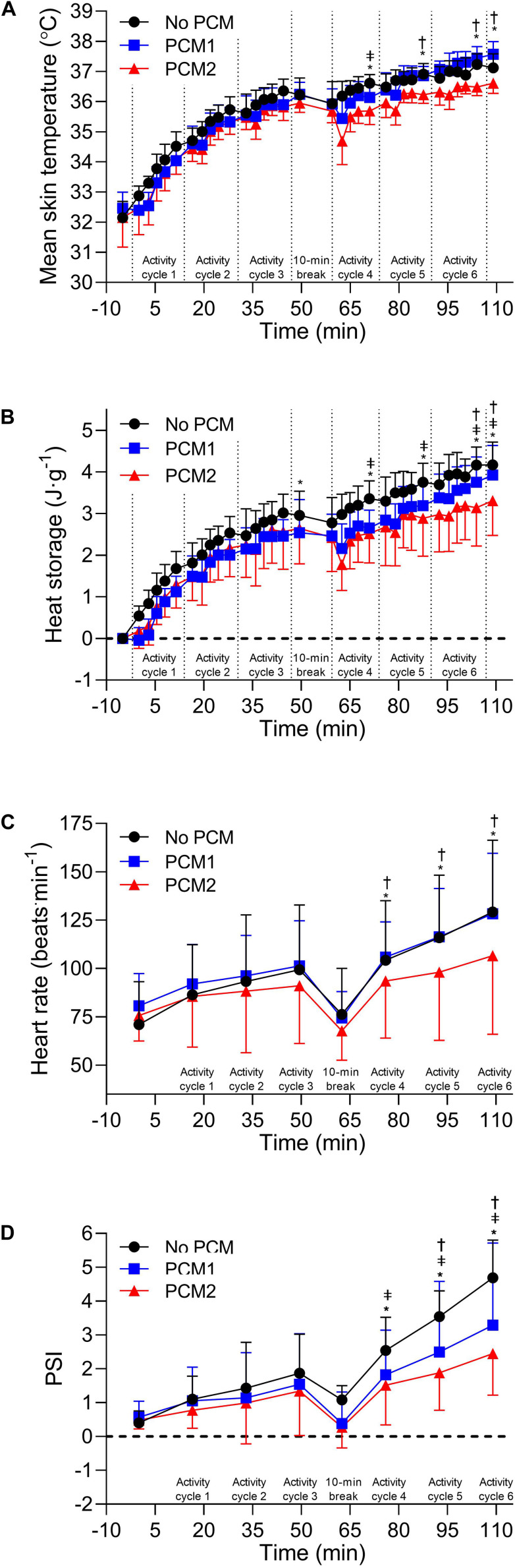
Mean skin temperature **(A)** and heat storage **(B)** recorded at baseline and 30 s before the end of the each exercise during an activity cycle, 10 min cooling period and 5 min rest periods. Measures recorded at baseline, during arm ergometry at the end at the end of each 16:30 min:s EOD activity cycle and at the end of a trial were compared between conditions (*N* = 5). Heart rate **(C)** and physiological strain index (PSI) **(D)** recorded at baseline and the mean heart rate over an activity cycle. Measures recorded at baseline and the mean heart rate over an EOD activity cycle were compared between conditions (*N* = 4). Data are the mean values ± 1 *SD.* Unidirectional error bars have been used to maintain clarity. *, difference between NoPCM and PCM2 (*p* < 0.05); ‡ = difference between NoPCM and PCM1 (*p* < 0.05); †, difference between PCM1 and PCM2 (*p* < 0.05).

There was a main effect for condition [*F*(2, 8) = 5.60, *p* = 0.030, η*_*p*_*^2^ = 0.583], time [*F*(7, 28) = 361.61, *p* < 0.001, η*_*p*_*^2^ = 0.989] and for an interaction between condition and time in T_*skin*_ [*F*(14, 56) = 4.87, *p* < 0.001, η*_*p*_*^2^ = 0.549]. *Post hoc* pairwise comparisons identified that T_*skin*_ was lower in PCM2 compared to NoPCM at the end of cycle 4 (by −0.93 [95% CI: −1.62, −0.23]°C, *p* < 0.001, *d*_*unb*_ = 1.97), cycle 5 (by −0.73 [95% CI: −1.34, −0.12]°C, *p* < 0.0001, *d*_*unb*_ = 2.07), cycle 6 (by −0.78 [95% CI: −1.45, −0.10]°C, *p* < 0.0001, *d*_*unb*_ = 2.12) and at end of the trial (by −0.61 [95% CI: −1.21, −0.10]°C, *p* = 0.048, *d_*unb*_* = 1.67). T_*skin*_ was lower in PCM2 compared to PCM1 at the end of cycle 5 (by −0.58 [95% CI: −0.88, −0.27]°C, *p* = 0.0002, *d_*unb*_* = 1.64), cycle 6 (by −0.90 [95% CI: −1.19, −0.61]°C, *p* < 0.0001, *d_*unb*_* = 2.40) and at the end of the trial (by −0.89 [95% CI: −1.37, −0.42]°C, *p* < 0.0001, *d_*unb*_* = 2.05). T_*skin*_ was also lower in PCM1 compared to NoPCM at the end of cycle 4 (by −0.44 [95% CI: −0.86, −0.01]°C, *p* = 0.0058, *d_*unb*_* = 1.40; Figure 3A).

There was a main effect for condition [*F*(2, 8) = 6.634, *p* = 0.020, η*_*p*_*^2^ = 0.624], time [*F*(7, 28) = 367.84, *p* < 0.001, η*_*p*_*^2^ = 0.989] and for an interaction between condition and time in heat storage [*F*(14, 56) = 3.96, *p* < 0.001, η***_*p*_***^2^ = 0.598]. *Post hoc* pairwise comparisons identified that heat storage was higher in NoPCM compared to PCM1 at the end of cycle 4 (by 0.80 [95% CI: 0.30, 1.31] J⋅g^–1^, *p* < 0.0001 = 0.012, *d_*unb*_* = 1.47) cycle 5 (by 0.67 [95% CI: 0.20,1.14] J⋅g^–1^, *p* = < 0.0001, *d_*unb*_* = 1.24), cycle 6 (by 0.52 [95% CI: 0.20,0.83] J⋅g^–1^, *p* = 0.0007, *d_*unb*_* = 0.91) and at the end of the trial (by 0.40 [95% CI: 0.08,0.71] J⋅g^–1^, *p* = 0.009, *d_*unb*_* = 0.62). Heat storage was higher in NoPCM compared to PCM2 at the end of cycle 3 (by 0.36 [95% CI: 0.05,0.68] J⋅g^–1^, *p* = 0.018, *d_*unb*_* = 0.45), cycle 4 (by 0.80 [95% CI: 0.49,1.12] J⋅g^–1^, *p* < 0.0001, *d_*unb*_* = 1.14), cycle 5 (by 0.86 [95% CI: 0.54,1.17] J⋅g^–1^), *p* < 0.0001, *d_*unb*_* = 0.92), cycle 6 (by 1.00 [95% CI: 0.69,1.32] J⋅g^–1^, *p* < 0.0001, *d_*unb*_* = 1.08), and the end of exercise (by 0.87 [95% CI: 0.55,1.18] J⋅g^–1^, *p* < 0.0001, *d_*unb*_* = 1.05). Heat storage was higher in PCM1 compared to PCM2 at the end of cycle 6 (by 0.49 [95% CI: 0.18,0.81] J⋅g^–1^), *p* = 0.0012, *d_*unb*_* = 0.50) and at the end of exercise (by 0.46 [95% CI: 0.15,0.78] J⋅g^–1^, *p* = 0.0023, *d_*unb*_* = 0.51); Figure 3B).

There was a main effect for time [*F*(5, 15) = 51.46, *p* < 0.001, η*_*p*_*^2^ = 0.995] and for an interaction between condition and time in the mean HR across an activity cycle over the six completed active cycles [*F*(10, 30) = 5.76, *p* < 0.001, η*_*p*_*^2^ = 0.658]. *Post hoc* pairwise comparisons identified that mean HR across a cycle was lower in PCM2 than PCM1 in activity cycle 4 (by −8 [95% CI: −21, −8] beats⋅min^–1^, *p* = 0.011, *d_*unb*_* = 0.38), cycle 5 (by −15 [95% CI: −22, − 7] beats⋅min^–1^; *p* < 0.0001; *d_*unb*_* = 0.50) and cycle 6 (by −17 [95% CI: −28,− 6] beats⋅min^–1^, *p* < 0.0001, *d_*unb*_* = 0.52) and lower in PCM2 than NoPCM in activity cycle 4 (by −11 [95% CI:-17,−4] beats.min^–1^, *p* = 0.0006, *d_*unb*_* = 0.43), cycle 5 (by −18 [95% CI: −24,−12] beats.min^–1^, *p* < 0.0001, *d_*unb*_* = 0.63), and cycle 6 (by −23. [95% CI: −29, −16] beats⋅min^–1^; *p* < 0.0001; *d_*unb*_* = 0.70; Figure 3C).

There was a main effect for condition [*F*(2, 6) = 6.99, *p* = 0.027, η*_*p*_*^2^ = 0.700], time [*F*(5, 15) = 79.43, *p* < 0.001, η*_*p*_*^2^ = 0.964) and for an interaction between condition and time in the mean PSI across an activity cycle over the six completed active cycles [*F*(10, 30) = 9.42, *p* < 0.001, η*_*p*_*^2^ = 0.758]. *Post hoc* pairwise comparisons identified that mean PSI across a cycle was lower in PCM1 than NoPCM in activity cycles 4 (by –0.7 [95% CI: −1.2,−0.3], *p* < 0.0011; *d_*unb*_* = 0.74), 5 (by −1.0 [95% CI: −1.5,−0.6], *p* < 0.0001; *d_*unb*_* = 0.79) and 6 (by –1.4 [95% CI: −1.8,−0.9], *p* < 0.0001, *d*_*unb*_ = 0.88). *Post hoc* pairwise comparisons identified that mean PSI across a cycle was lower in PCM2 than NoPCM in activity cycles 4 (by −1.0 [95% CI: −2.0,−0.1], *p* = 0.00014; *d_*unb*_* = 1.14), 5 (by −1.7 [95% CI: −2.2,−1.1], *p* = 0.00013; *d_*unb*_* = 2.08) and 6 (by −2.2 [95% CI: −3.1,−1.4], *p* ≤ 0.00014, *d*_*unb*_ = 2.26). Mean PSI was also lower in PCM2 compared to PCM1 in activity cycles 5 (by −0.6 [95% CI: –1.1,−0.2], *p* = 0.005, *d*_*unb*_ = 0.44) and cycle 6 (by −0.8 [95% CI: −1.3,−0.4], *p* = 0.0002, *d*_*unb*_ = 0.52; Figure 3D).

Differences in sweat rates during NoPCM (1.42 [95% CI: 0.71, 2.12] L.h^–1^), PCM1 (1.22 [95% CI: 0.53, 1.90] L.h^–1^) and PCM2 (0.90 [95% CI: 0.44, 1.37] L.h^–1^) did not meet conventional levels of statistical significance (all *p* > 0.05).

### Subjective Measurements

**FIGURE 4:**
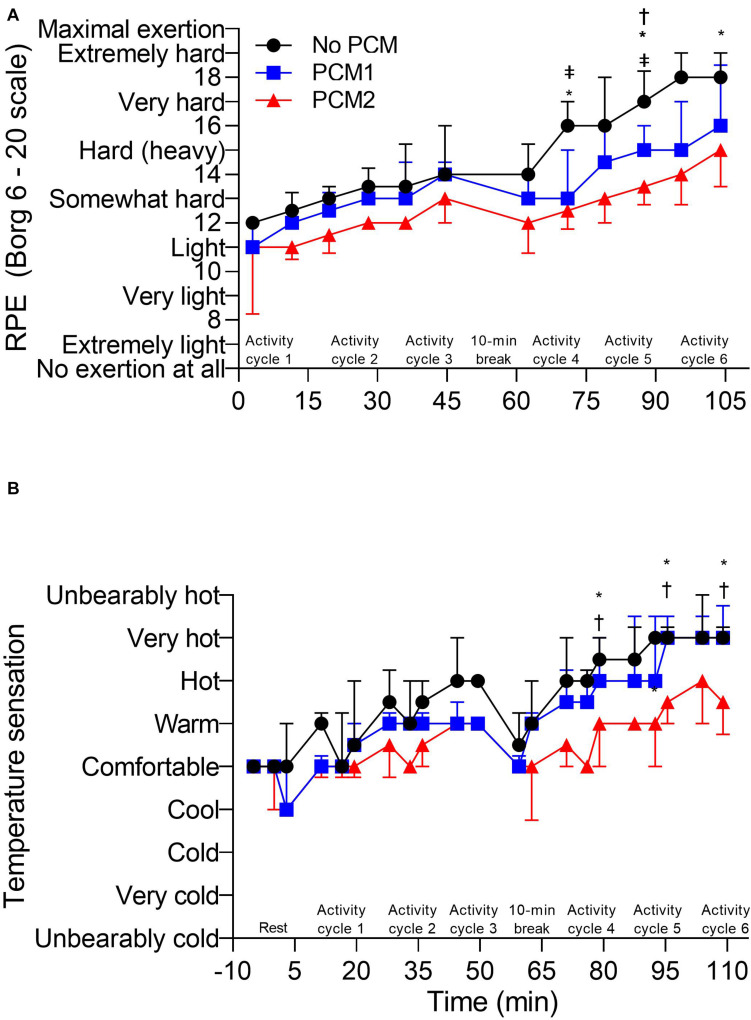
**(A)** Rating of perceived exertion (RPE) recorded at the end of the treadmill walking and arm ergometry exercise of each activity cycle. **(B)** Temperature sensation (TS) recorded at baseline and 30 s before the end of the each exercise during an activity cycle, 10 min cooling period and 5 min rest periods. Rating of perceived exertion recorded during arm ergometry at the end at the end of each 16:30 min:s EOD activity cycle were compared between conditions (*N* = 5) and TS recorded at baseline and during arm ergometry at the end at the end of each 16:30 min:s EOD activity cycle were compared between conditions (*N* = 5). NB Thermal comfort follows a similar pattern to TS. Data are the median values ± interquartile range. Unidirectional error bars have been used to maintain clarity. *, difference between NoPCM and PCM2 (*p* < 0.05); ‡ = difference between NoPCM and PCM1 (*p* < 0.05); †, difference between PCM1 and PCM2 (*p* < 0.05).

There was a main effect for condition [*F*(2, 8) = 8.22, *p* = 0.011, η*_*p*_*^2^ = 0.673], time [*F*(5, 20) = 103.27, *p* < 0.001, η*_*p*_*^2^ = 0.963] and for an interaction between condition and time in RPE [*F*(10, 40) = 3.32, *p* = 0.003, η*_*p*_*^2^ = 0.454]. *Post hoc* pairwise comparisons identified that RPE was lower in PCM2 compared to NoPCM at the end of activity cycle 4 (by −3.0 [95% CI: −4.2, −1.8], *p* < 0.0001, *d_*unb*_* = 1.61), 5 (by −3.2 [95% CI: −4.2, −2.2], *p* < 0.0001, *d_*unb*_* = 1.95) and 6 (by −3.4 [95% CI: −5.0, −1.7], *p* < 0.0001, *d_*unb*_* = 1.93), and lower compared to PCM1 after activity cycles 5 (by −1.6 [95% CI: −2.9, −0.3], *p* = 0.015, *d_*unb*_* = 0.86) and 6 (by −2.4 [95% CI: −3.7, −1.1], *p* = 0.0002, *d_*unb*_* = 1.09). RPE was lower in PCM1 compared to NoPCM at the end of activity cycle 4 (by −2.4 [95% CI: −4.3, −0.5], *p* = 0.0002, *d_*unb*_* = 1.21) and activity cycle 5 (by −1.6 [95% CI: −2.9, −0.3], *p* = 0.015; Figure 4A).

There was a main effect for condition [*F*(2, 8) = 12.70, *p* = 0.003, η*_*p*_*^2^ = 0.760], time [*F*(6, 24) = 45.17, *p* < 0.001, η*_*p*_*^2^ = 0.919], and for an interaction between condition and time in temperature sensation [*F*(12, 48) = 2.10, *p* = 0.035, η*_*p*_*^2^ = 0.344]. *Post hoc* pairwise comparisons identified that temperature sensation was lower in PCM2 compared to NoPCM at the end of activity cycles 4 (by −1.8 [95% CI: −2.8,−0.8], *p* = 0.009, *d_*unb*_* = 1.90), 5 (by −1.0 [95% CI: −1.9,−0.1], *p* = 0.034, *d_*unb*_* = 1.49), and 6 (by −1.4 [95% CI: −2.1,−0.7], *p* = 0.005, *d_*unb*_* = 1.25). Temperature sensation was also lower in PCM2 compared to PCM1 at the end of activity cycle 4 (by −1.4 [95% CI: −2.5,−0.3], *p* = 0.006, *d_*unb*_* = 1.36), cycle 5 (by −1.2 [95% CI: −1.9, −0.5], *p* = 0.0008) and cycle 6 (by −1.2 [95% CI: −1.9, −0.45], *p* = 0.0008; Figure 4B).

There was a main effect for condition [*F*(2, 8) = 13.41, *p* = 0.003, η*_*p*_*^2^ = 0.770], time [*F*(6, 24) = 48.08, *p* < 0.001, η*_*p*_*^2^ = 0.923], and for an interaction between condition and time in thermal comfort [*F*(12, 48) = 3.30, *p* = 0.002, η*_*p*_*^2^ = 0.452]. Similar to RPE, *post hoc* pairwise comparisons identified that thermal comfort was lower in PCM2 compared to NoPCM at the end of activity cycle 4 (by −1.8 [95% CI: −2.8, −0.8], *p* = 0.00019, *d_*unb*_* = 1.90), cycle 5 (by −1.6 [95% CI: −2.7,−0.5], *p* = 0.00012, *d_*unb*_* = 1.78) and cycle 6 (by −1.6 [95% CI: −2.3,−0.9], *p* = 0.00013, *d_*unb*_* = 1.39), and lower than PCM1 at the end of activity cycle 4 (by −1.0 [95% CI: −1.7,−0.3], *p* = 0.0028, *d_*unb*_* = 1.37), cycle 5 (by −1.4 [95% CI: −2.1,−0.7], *p* < 0.0001, *d_*unb*_* = 1.48) and cycle 6 (by −1.2 [95% CI: −1.9,−0.5], *p* = 0.0003, *d_*unb*_* = 1.16). Thermal comfort was also lower in PCM1 compared to NoPCM in cycle 2 (by −0.8 [95% CI: −1.4, −0.2], *p* = 0.019, *d_*unb*_* = 1.19; Table 1).

**TABLE 1:** Physiological (mean, 95% CI) and perceptual responses (median, interquartile range) at the end of the last simulated EOD operator activity cycle (i.e., cycle 6) and at the end of the trial.

	T_*re*_ (°C)		T_*skin*_ (°C)		HR (beats⋅min^–1^)		T_*re*_-T_*skin*_ (°C)		PSI		PeSI		RPE		TC		TS	
									
	Cycle 6	End	Cycle 6	End	Cycle 6	End	Cycle 6	End	Cycle 6	End	Cycle 6	End	Cycle 6	End	Cycle 6	End	Cycle 6	End
NoPMC	38.01 (37.8, 38.4)	38.11 (37.8, 38.6)	37.32 (37.0, 37.7)^‡^	37.29 (37.0, 37.6)*	129 (92, 166) ‡	–	0.70 (0.31,1.1)	0.83 (0.58, 1.1)	4.7 (3.6, 5.8)	–	8.3 (7.5 to 8.7)	–	17.0 (16.5 to 19.0)	–	8.0 (6.5 to 8.0)	7.0 (6.5 to 8.0)	7.0 (6.5 to 8.0)	7.0 (6.0 to 7.5)

PMC1	37.90 (37.6, 38.2)	38.01 (37.7, 38.3)^¥^	37.44 (37.1, 37.8)	37.57 (37.2, 38.0)	124 (105, 162)	–	0.46 (0.01,0.91)	0.43 (0.10, 0.76)	3.3 (0.9, 5.7)	–	7.4 (6.3 to 8.5)	–	16.0 (13.0 to 15.0)	–	7.0 (6.5 to 7.5) ¥	7.0 (6.5 to 7.5)	7 (6.5 to 7.5)¥	7.0 (6.3 to 7.8)

PMC2	37.41 (36.7, 38.1)	37.54 (37.1, 38.0)^‡^	36.54 (36.2, 36.9)^¥^	36.68 (36.3, 37.1)^¥^	107 (91, 145) ^¥^	–	0.89 (0.10, 1.64)	0.87 (0.30, 1.43)	2.5 1.2, 3.7)^‡^	–	6.3 (5.5 to 6.6)^†^	–	15.0 (13.0 to 15.0)‡	–	6.0 (5.0 to 6.5)^‡^	6.0 (5.0 to 6.5)	6.0 (5.0 to 6.5)†	6.0 (5.0 to 6.5)

*, NoPCM vs. PCM2 ≤ 0.05; †, NoPCM vs. PCM2 ≤ 0.01; ‡, NoPCM vs. PCM2 ≤ 0.001; † = PCM1 vs. PCM2 ≤ 0.05; ¥, PCM1 vs. PCM2 ≤ 0.001. T_re_, rectal temperature; T_skin_, skin temperature; HR, heart rate; PSI, physiological strain index; PeSI, perceptual-based strain index; RPE, rating of perceived exertion; TC, thermal comfort; TS, temperature sensation. Measurements from the end of unloaded-arm ergometry were compared in activity cycle 6. Measurements collected at the end of the cycle 6 rest period are the end of trial measurements (Figure 1).

There was a main effect for condition [*F*(2, 8) = 17.66, *p* = 0.001, η*_*p*_*^2^ = 0.815], time [*F*(5, 20) = 3.30, *p* = 0.025, η*_*p*_*^2^ = 0.452] for PeSI but no interaction between condition and time. Similar to RPE and TC, *post hoc* pairwise comparisons identified that PeSI was lower in PCM2 compared to NoPCM at the end of activity cycles 4 (by −1.7 [95% CI: −3.13, −0.21], *p* = 0.034, *d_*unb*_* = 0.80), 5 (by −1.8 [95% CI: −2.8, −0.8], *p* = 0.003, *d_*unb*_* = 1.56) and 6 (by −2.0 [95% CI: −3.2, 0.8], *p* = 0.010, *d_*unb*_* = 1.94; Table 1).

## Discussion

These data demonstrate that replacing a PCM cooling vest after ∼50 min of simulated EOD operator activity when wearing an EOD suit can extend the efficacy of PCM per cooling to attenuate increases in heat strain and produce more favorable thermal perceptions; both of which could potentially increase work tolerance times (Bach et al., 2019). Either during or at the end of the second period of simulated EOD operator activity (∼60–109 min), indices of physiological strain were lower in the trials where the PCM cooling vest was replaced (PCM2) compared to wearing either no PCM cooling vest (NoPCM) or when the PCM cooling vest is not replaced (PCM1). This reduction in physiological strain is reflected in PSI, with the average PSI being lower in PCM2 vs. NoPCM and PCM1 in the second period of simulated EOD operator activity. In the second period (the final three simulated EOD operator activity cycles), rectal temperature increased at a similar rate in PCM1 to NoPCM to reach a similar value at the end of the trial. This indicates that once the PCM in the vest has changed phase it becomes a further layer of insulation, negatively affecting thermoregulation, and providing no further benefit to the operator. More favorable perceptions (i.e., RPE, TS, and TC) were also experienced in PCM2 in the second period of simulated EOD operator activity in comparison to NoPCM and PCM1.

Phase change material cooling garments have been shown to be both effective (McLellan and Frim, 1998; Chou et al., 2008; House et al., 2013; Maley et al., 2020), and ineffective (Cleary et al., 2014; Eijsvogels et al., 2014) in attenuating heat strain. This contradiction appears to be related to several factors that affect the ability of PCM based cooling garments to attenuate physiological strain such as ambient temperature (Muir et al., 1999), intensity of exercise (Smolander et al., 2004; Eijsvogels et al., 2014), BSA coverage of the PCM (Gao et al., 2010), melting temperature of the PCM (House et al., 2013), mass of the PCM garment (Gao et al., 2010) and the cooling regime used, i.e., either pre-cooling, intermittent cooling (only used during rest periods) or per-cooling (continuous cooling) (Cleary et al., 2014; Maley et al., 2020). Of these factors it appears that PCM cooling garments are most effective in heat stress exposures where: (1) evaporative heat loss is limited (such as when PPE is worn); (2) blood flow to the skin is not impaired (House et al., 2013); (3) the PCM covers a large BSA without compromising mobility (Gao et al., 2010); and (4) when the temperature gradient between the PCM melting temperature and the skin temperature is equal or greater than 6°C (Gao et al., 2010). Nonetheless, in their meta-analysis, Chan et al. (2015) identified that in general, vs. no cooling, PCM cooling garments can attenuate rises in core temperature by 0.43°C.h^–1^ and sweat rate by 0.24 L.h^–1^, both of which are slightly lower than that observed in the current study when the PCM cooling vest was replaced (i.e., −0.49°C.h^–1^ and −0.52 L.h^–1^ in rectal temperature and sweat rate, respectively). Chan et al. (2015) also identified that personal cooling garments as a collective reduce heart rate and mean skin temperature by 11 beats⋅min^–1^ and 0.67°C.h^–1^, respectively; values similar to what was observed in HR, but slightly higher than observed in skin temperature in the present study (i.e., 17 beats⋅min^–1^ and 0.32°C.h^–1^, respectively). However, even if the conditions conducive to increasing the efficacy of a PCM cooling garment are met, the benefit of the PCM garment is short lived, lasting between 45 and 120 min (Chan et al., 2015). This time course is dependent upon on the amount of heat either produced by the wearer or the external environment exhausting the cooling capacity sooner rather than later.

This acknowledged limitation in the cooling capacity of PCM garments is demonstrated in the present study. When the PCM cooling vest is not replaced after ∼50 min of simulated EOD operator activity (PCM1), by the end of the heat exposure (i.e., 109 min of simulated EOD activity) the PCM cooling vest provided no extra benefit to the user as the majority of physiological and thermal perceptual responses were similar to when no PCM cooling vest was worn. In addition, Figure 2A suggests that, if the length of simulated EOD operator activity was extended, not replacing the PCM cooling vest could, over time, become detrimental to the user. For example, all participants completed all the trials with exception of one participant in the condition when the PCM cooling vest was not replaced. This is because, over time, core temperature could exceed that experienced when no PCM cooling vest is worn. As the metabolic rate, clothing and environmental parameters are similar in each condition, the additional insulation and non-functional mass provided by the PCM cooling vest is the likely cause of the increased rise in rectal temperature experienced in PCM1 during the 2nd period of simulated EOD operator activity.

As previously highlighted, by the end of the heat exposure, rectal temperature is similar in PCM1 and NoPCM (i.e., ∼38.0°C). However, replacing the PCM cooling vest during the 10 min rest period (PCM2) attenuates this rise resulting in an end core temperature of ∼37.5°C. The importance of this attenuation in core temperature is 2-fold. Firstly, to prevent heat associated illnesses in thermally stressful occupations, several occupational heat stress standards or guidelines use a core temperature limit of 38.0°C (as a group average) to ensure that the majority of workers do not reach core temperatures generally associated with hyperthermia-induced fatigue (i.e., 38.5–39.5°C) and fatal heat illnesses, such as heat stroke (i.e., 42°C) (e.g., International Organization for Standardization (ISO) 7993, 2004; National Institute for Occupational Safety and Health (NIOSH), 2016). Secondly, reduced work tolerance times generally correspond with lower core temperatures (e.g., 37.8–38.2°C) and higher skin temperatures (e.g., 37.8–38.2°C) when encapsulating PPE is worn (such as an EOD suit) as opposed to non-encapsulating PPE (Montain et al., 1994; Stewart et al., 2014; McLellan and Havenith, 2016); reducing the thermal gradient between core and skin temperature required for optimal heat loss. Therefore, the observed ∼0.5°C attenuation in core temperature may protect the majority of EOD operators from succumbing to hyperthermia-induced fatigue or thermal injury, which otherwise could compromise the performance and health and safety of the operator.

Replacing the PCM cooling vest reduced cardiovascular strain compared to PCM1 and NoPCM causing an overall reduction in physiological strain as reflected in the physiological strain index (PSI). The PSI was developed by Moran et al. (1998b) to reduce incidences of heat-related illnesses during exercise in hot conditions at an individual level. The PSI has been validated to be able to distinguish different levels of heat strain in different heat stress scenarios including when PPE is worn (Moran et al., 1998a, 1999; Moran, 2000; Petruzzello et al., 2009). A PSI value of ≥6 is considered as the “high physiological strain zone” and a value of ≥7.5 is considered as being at high risk for thermal injury (Moran et al., 1998a; Buller et al., 2008). However, at an individual level, PSI values lower than 4 have been associated with thermal intolerance in heat stress scenarios where encapsulated PPE is worn whilst performing moderate continuous exercise in the heat (Stewart et al., 2014).

Due to the intermittent nature of exercise, PSI in the present study was calculated from HR averaged across an activity cycle. Replacing the PCM cooling vest maintained a lower PSI value by the end of the last activity cycle in the second period of simulated EOD operator activity (i.e., 2.5) compared to PCM1 (i.e., 3.2) and NoPCM (i.e., 4.7). During the most physiological demanding task where the highest heart rates were achieved (i.e., crawling), in the last activity cycle (cycle six), PSI was lower in PCM2 (i.e., 2.9, 3/5 participants below 2.9) than PCM1 (i.e., 4.1, 1/5 participants below 2.9) and NoPCM (i.e., 5.1, 0/5 participants below 2.9), indicating that replacing the PCM cooling vest could possibly extend work tolerance and reduce health risks for the operator. However, it is acknowledged that, due to the type of exercise being different between studies such as Stewart et al. (2014) (i.e., continuous fixed-paced exercise of one intensity vs. intermittent exercise that varies in the level of intensity and includes rest periods), caution must be taken when comparing PSI values associated with thermal intolerance. The intermittent exercise used in the current study provides a more dynamic thermal environment and it is currently not understood how this may effect PSI thresholds associated with thermal intolerance.

It has been suggested that the perception-based heat strain index (PeSI) developed by Tikuisis et al. (2002) provides a better or equivalent indicator of risk of thermal intolerance than the PSI, including when PPE (such as EOD suits) is worn (Tikuisis et al., 2002; Petruzzello et al., 2009; Borg et al., 2017). In Borg et al. (2017) participants completed up to 120 min of continuous treadmill walking in different wet bulb globe temperatures (WBGT), at different exercise intensities, whilst wearing different types of PPE, including EOD suits. Across the different conditions, thermal intolerance was experienced at a PeSI value of 6.2 (at a group level), a value similar to that observed in Tikuisis et al. (2002), i.e., 6.5. The improved ratings of temperature sensation and perceived exertion experienced in PCM2 caused PeSI to be lower in PCM2 (i.e., 6.1, 4/5 participants below 6.5) than NoPCM (i.e., 8.1, 0/5 participants below 6.5) and also lower, yet did not meet conventional thresholds for statistical significance, than PCM1 (i.e., 7.4, 1/5 participants below 6.5). Even though we must take into consideration that more studies are required to determine the thresholds in PeSI associated with thermal intolerance (especially in heat exposures that involve intermittent exercise), replacing the PCM cooling vest maintained the mean PeSI value below 6.5. Whereas a mean PeSI value of 7 (a threshold that could be considered to distinguish between low-moderate vs. high strain; Borg et al., 2017), was exceeded in both PCM1 and NoPCM. These observations suggests replacing the PCM cooling vest has the potential to reduce both physiological and thermal perceptual strain for the majority of EOD operators.

Based on the known limitation of PCM cooling garments regarding the time period of its effectiveness, replacing a PCM cooling garment during operation sounds quite an obvious thing to do to extend work tolerance and to protect personnel from hyperthermia-induced fatigue or thermal injury. However, the ability the recharge and/or replace a PCM cooling garment without removing the PPE during operation might be why this strategy has yet to be widely adopted into practice. To combat this issue Muir et al. (1999) adapted an impermeable PPE encapsulating suit (total mass: ∼6 kg) to allow six frozen ice gel packs (20 × 15 cm, mass: 2.2 kg) to be inserted into pockets outside the PPE, which were held tightly to the torso region by Velcro straps. This adaption made the frozen packs easily accessible to be replaced. Compared to no cooling, tolerance times during 2 h of moderate continuous exercise were extended by 9, 71, and 88% in 18, 23, and 28°C WBGT, respectively. Unfortunately, a control condition, were the ice packs were not replaced, was not present, therefore, the efficacy of replacing the ice packs every 30 min is unknown. Regardless, future designs of EOD suits might want to consider incorporating a similar approach, if the design does not jeopardize protection.

In a similar study design to the present study, but with a lighter EOD suit ∼21 kg, replacing the PCM cooling vest during the 10 min rest period, was as effective as that observed in the current study in reducing physiological and perceptual strain compared to wearing no PCM cooling vest. However, not replacing the PCM cooling vest after ∼50 min of simulated EOD operator activity could be considered less detrimental to the user. By the end of 109 min of simulated EOD operator activity, core temperature was lower in both trials where a PCM cooling vest was either worn throughout (37.7°C) or replaced (37.5°C), compared to when no PCM cooling vest was worn (38.0°C) (Unpublished data). It is likely that in the lighter EOD suit, the PCM cooling vest’s cooling capacity is extended with regards to duration, as the metabolic rate of the wearer is lower than when the heavier suit is worn. Similarly, the cooling method adopted in Muir et al. (1999) was not as effective in the coolest ambient condition (18°C WBGT) where the level of heat stress experienced by the participants would be less. Both of these observations highlight that the decision to replace a PCM cooling garment or not, or when to replace a PCM cooling garment, will be highly dependent upon the heat stress scenario the EOD operator is being exposed to.

There are some limitations to this study. The sample size of the study and the population used (i.e., young males) compromises the translation of the findings to a wider population, especially as the population of EOD operators is becoming more diverse, e.g., a wider age range and inclusion of both sexes. Therefore, further studies are required to confirm these initial findings, especially in more diverse populations that are representative of EOD operators. In the present study, the effect of replacing the PCM cooling vest was only assessed in one environmental condition, therefore, it would be of interest to examine the effect of replacing the PCM cooling vest in multiple heat stress scenarios that EOD operators are likely be exposed to. Currently it is also not known how replacing the PCM cooling vest could extend work tolerance as the period of simulated EOD operator activity was fixed. Cooling other parts of the body, especially those that maybe more accessible, may also be worth investigating with regards to extending thermal tolerance when wearing EOD suits. Finally, cognitive measures were not assessed in this study, therefore the question still remains whether replacing the PCM cooling vest can influence cognitive ability; a factor which is important with regards to both work performance and safety. Future investigations should be focus on addressing these limitations.

## Conclusion

In summary, the findings from the present exploratory study suggest that replacing a PCM cooling vest worn underneath an EOD suit better attenuates rises in both physiological and perceptual strain compared to when a PCM cooling vest is not replaced. In addition, the results suggest that continuing to wear a PCM cooling vest that has changed phase, i.e., exhausted its cooling capacity, increases the level of heat strain experienced by the wearer. Therefore, it is better to remove the vest at this point, or simply not to wear one at all, to reduce the probability of the wearer experiencing hyperthermia-induced fatigue or thermal injury. Further studies are required to confirm these initial findings especially in other environmental conditions that EOD operators are likely to be exposed to.

## Data Availability Statement

The raw data supporting the conclusions of this article will be made available by the authors, without undue reservation, to any qualified researcher.

## Ethics Statement

The studies involving human participants were reviewed and approved by the Coventry University Ethics Committee. The patients/participants provided their written informed consent to participate in this study.

## Author Contributions

CT, MO, and MS designed the study. MS and CT collected the data. SD, BL, and MS analyzed the data. SD, BL, and CT undertook the data interpretation and manuscript preparation. All authors approved the final version of the manuscript.

## Conflict of Interest

The authors declare that this study received funding from NP Aerospace Ltd., Coventry, United Kingdom. The funder provided the EOD equipment and funded MS’s Mres project. MO also provided user specific knowledge to assist with the study design.
